# Supplementary material for: Enhancing NK cell-mediated tumor killing of B7-H6+ cells with bispecific antibodies targeting allosteric sites of NKp30
Source: Mol Ther Oncol. 2024 Dec 6;33(1):200917. doi: 10.1016/j.omton.2024.200917 (PMC11730255; doi:10.1016/j.omton.2024.200917)
Supplement: Document S1. Figures S1 and S2 and Table S1 [file mmc1.pdf]

OMTON, Volume *33*

Supplemental information

Enhancing NK cell-mediated tumor killing of B7-H6^{+} cells with bispecific antibodies targeting allosteric sites of NKp30

Léxane Fournier, Paul Arras, Lukas Pekar, Harald Kolmar, Stefan Zielonka, Lars Toleikis, and Stefan Becker

Table S1: Analytics and Yields of a-NKp30 VHH-Fc fusion. Expression yields were determined after protein purification. Purity was determined by analytic size-exclusion chromatography (SEC). HEK: Human Embryonic Kidney. CHO: Chinese Hamster Ovaries. ND: non-determined

Antibody	Yield HEK (mg/L)	SEC HEK (%)	Yield CHO (mg/L)	SEC CHO (%)
A10	ND	ND	ND	ND
B4	220.5	100	201.1	95.5
B6	121.4	98.8	39.7	91.1
B10	141.6	77.5	47.5	96.7
D10	211.9	98.9	210.1	90.2
E4	268.5	100	242.3	100
E11	287.6	100	297.5	100
G5	271.9	100	249.8	100
G10	122.1	100	54.8	100

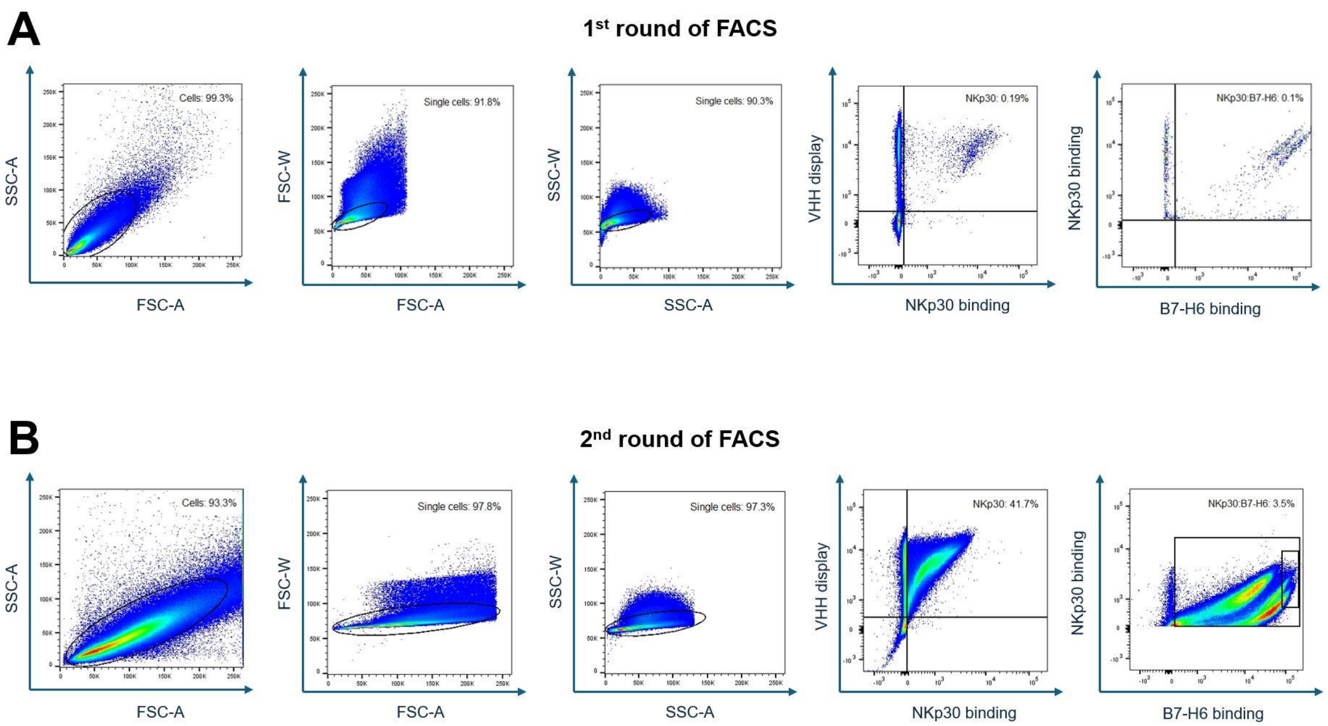

Figure S1: Gating strategy for the sorting of non-B7-H6 competitive NKp30-binders.

The yeast surface display library was screened by FACS. The first gate (FSC-A vs SSC-A) was designed to select yeast cells. From these cells, we selected single cells first based on the plot FSC-A vs FSC-W, and secondly from a gate SSC-A vs SSC-W. From these single cells, a two-dimensional gate to identify functional VHH display in combination to NKp30 binding. From these NKp30 binders, a second two-dimensional gate was applied to select NKp30 binders that were not competing with B7-H6. From the first round of sorting 0.19% of the library were binding NKp30 while 0.1 % were binding NKp30 in the presence of B7-H6. These 0.1% were selected for sorting. For the second and final round, 41.7% of the library were NKp30 binders. We applied a more stringent sorting gate to select VHHs that were better positively modulating NKp30 affinity for B7-H6 and selected 3.5% of the library for sorting.

Applied sorting gates and corresponding cell populations (as % of total cells) are shown. Plots were generated using FlowJo™.

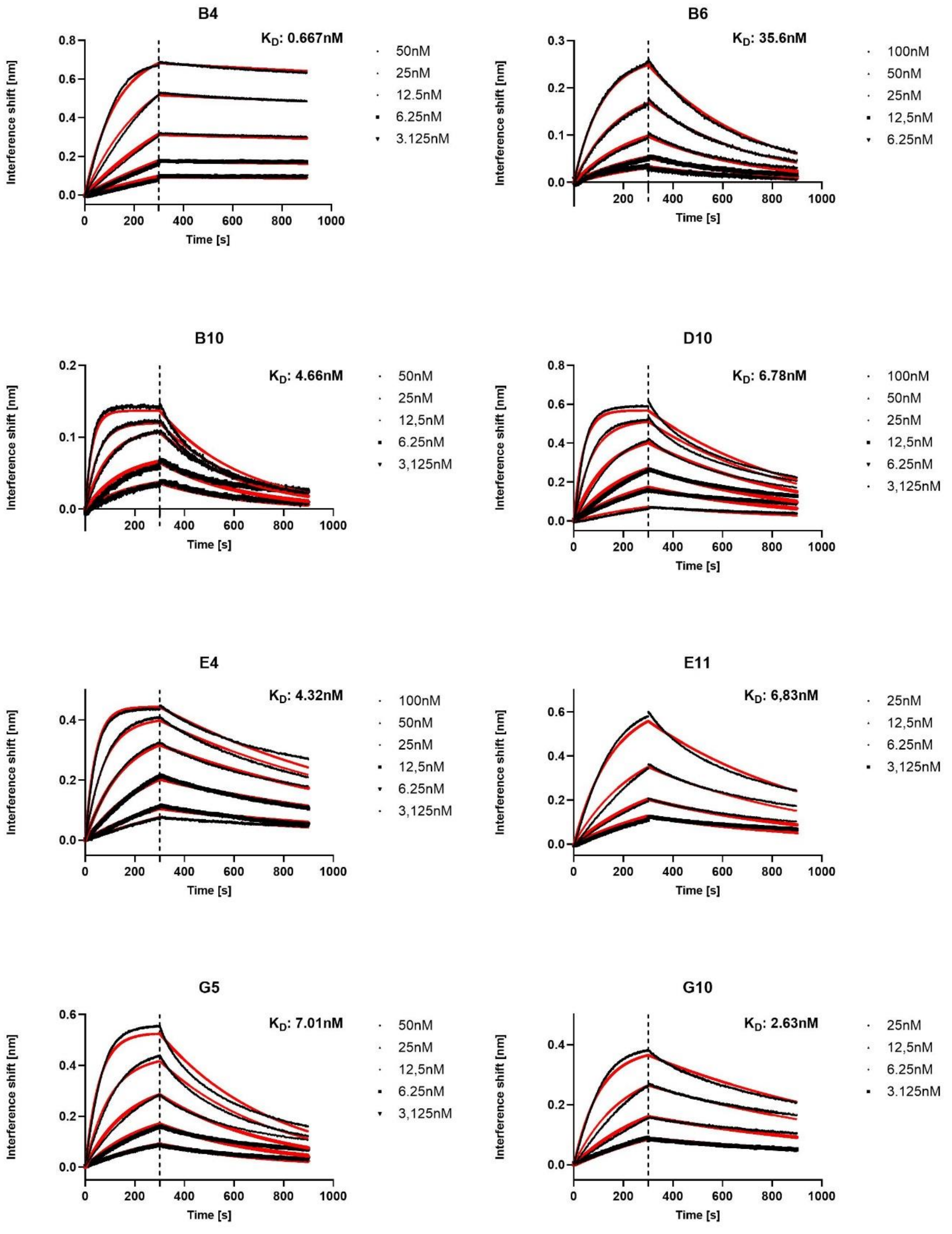

Figure S2: K_D determination of anti-NKp30 for rhNKp30. The experiment was performed by BLI. Data were fitted to a 1:1 model (fitting in red).
