# Supplementary material for: Enhancing NK cell-mediated tumor killing of B7-H6+ cells with bispecific antibodies targeting allosteric sites of NKp30
Source: Mol Ther Oncol. 2024 Dec 6;33(1):200917. doi: 10.1016/j.omton.2024.200917 (PMC11730255; doi:10.1016/j.omton.2024.200917)
Supplement: Document S2. Article plus supplemental information [file mmc2.pdf]

Molecular Therapy
Oncology
Original Article

Enhancing NK cell-mediated tumor killing of B7-H6$^+$ cells with bispecific antibodies targeting allosteric sites of NKp30

Léxane Fournier,[1,2] Paul Arras,[3] Lukas Pekar,[3] Harald Kolmar,[2,4] Stefan Zielonka,[2,3] Lars Toleikis,[1] and Stefan Becker[1]

[1]Early Protein Supply and Characterization, Merck Healthcare KGaA, 64293 Darmstadt, Germany; [2]Institute for Organic Chemistry and Biochemistry, Technical University of Darmstadt, 64287 Darmstadt, Germany; [3]Antibody Discovery and Protein Engineering, Merck Healthcare KGaA, 64293 Darmstadt, Germany; [4]Centre for Synthetic Biology, Technical University of Darmstadt, 64283 Darmstadt, Germany

In this work, we report the discovery and engineering of allosteric variable domains of the heavy chain (VHHs) derived from camelid immunization targeting NKp30, an activating receptor on natural killer (NK) cells. The aim was to enhance NK cell-mediated killing capacities by identifying VHHs that do not compete with the natural ligand of NKp30:B7-H6, thereby maximizing the recognition of B7-H6$^+$ tumor cells. By relying on the DuoBody technology, bispecific therapeutic antibodies were engineered, creating a panel of bispecific antibodies against NKp30xEGFR (cetuximab moiety) or NKp30xHER2 (trastuzumab moiety), called natural killer cell engagers (NKCEs). These NKCEs were assessed for their killing capacities on B7-H6-expressing tumor cells. The results demonstrated an enhancement in NK killing capacities for both EGFR-expressing (HeLa) and HER2-expressing (SK-BR-3) cells, indicating the significance of the natural NKp30/B7-H6 axis in tumor recognition by the immune system. Notably, engineering NKCEs to allow natural recognition of B7-H6 was found to be more effective in promoting NKCE-mediated killing of B7-H6$^+$ tumor cells via enhancement of cytokine release. This study highlights the potential of an enhanced-targeting approach, wherein tumor cell surface antigens are targeted while still enabling the natural recognition of the activating ligand (B7-H6) by the immune cells.

INTRODUCTION

Natural killer (NK) cells are key players of the innate immune system acting as an early defense against pathogens or stressed or tumor cells.[1,2] An essential feature of NK cells is their ability to distinguish stressed or tumor cells and contribute to their elimination.[2,3] The NK-mediated cell lysis response depends on overly complex mechanisms not fully elucidated but involving an interplay of activating and inhibitory receptors.[2,3] Notably, inhibitory receptors such as NKG2A, inhibitory killer cell immunoglobulin (Ig)-like receptors , and T cell immunoreceptor with Ig and tyrosine-based inhibitory motif domain recognize safe ligands associated as self-markers.[2,3] However, activating receptors include NKG2D, activating KIRs, DNAM-1, and the natural cell cytotoxicity receptors (NCRs)[4,5] NKp30,[6] NKp44, and NKp46. These receptors can recognize a diverse array of ligands mainly upregulated during tumor progression, such as MICA/B, CD155, B7-H6,[7] or heparan sulfate.[8] Intriguingly, the shedding of these ligands by metalloproteases has been associated with the inhibitory effects of soluble ligands, enabling tumor escape.[9,10]

Given the critical role of tumor cell recognition, strategies to maintain or boost NK cell detection of tumors may hold great promise for the development of novel therapeutic approaches.[11,12] Thus, efforts have been made to use biologics as new modalities to approach more intricate mechanisms of action by redirecting the immune system. Emerging strategies rely on engineering antibodies that can simultaneously target a tumor-associated antigen and a cell surface receptor of NK cells to facilitate antitumor activity by bringing immune cells in close proximity to tumor cells.[12,13] Innate Pharma recently out-licensed NK cell engager (NKCE) clinical candidates to Sanofi based on their platform ANKET (antibody-based NKCEs therapeutics), which is one of the most advanced programs for NKCEs.[13,14]

Several groups have been working on NKCEs by targeting, notably, the NCRs such as NKp46[14–16] or NKp30.[17–20] The latest one has been shown to successfully engage and redirect NK cells to elicit tumor cell lysis of epidermal growth factor receptor-positive (EGFR$^+$) cells in multiple studies.[17–20] Our group showed that lowest half-maximal effective concentration (EC_{50}) values were achieved in a targeted manner for B7-H6 competitive NKCE, and the prominent maximum killing effects were achieved with an NKCE that was not competing with B7-H6.[18,20] However, these effects were solely tested on B7-H6$^-$ tumor cells (A431 and A549 cells).[9] We hypothesize that engineering NKCEs that do not compete with B7-H6 for binding to NKp30 will enhance the killing properties against B7-H6$^+$ tumor cells. This will be possible through the natural recognition axis NKp30/B7-H6, which acts as a danger signal and provides an

Received 21 August 2024; accepted 3 December 2024;
https://doi.org/10.1016/j.omton.2024.200917.

Correspondence: Stefan Becker, Early Protein Supply and Characterization, Merck Healthcare KGaA, Frankfurter Strasse 250, 64293 Darmstadt, Germany
E-mail: stefan.c.becker@merckgroup.com

Published by Elsevier Inc. on behalf of The American Society of Gene and Cell Therapy.
This is an open access article under the CC BY license (http://creativecommons.org/licenses/by/4.0/).

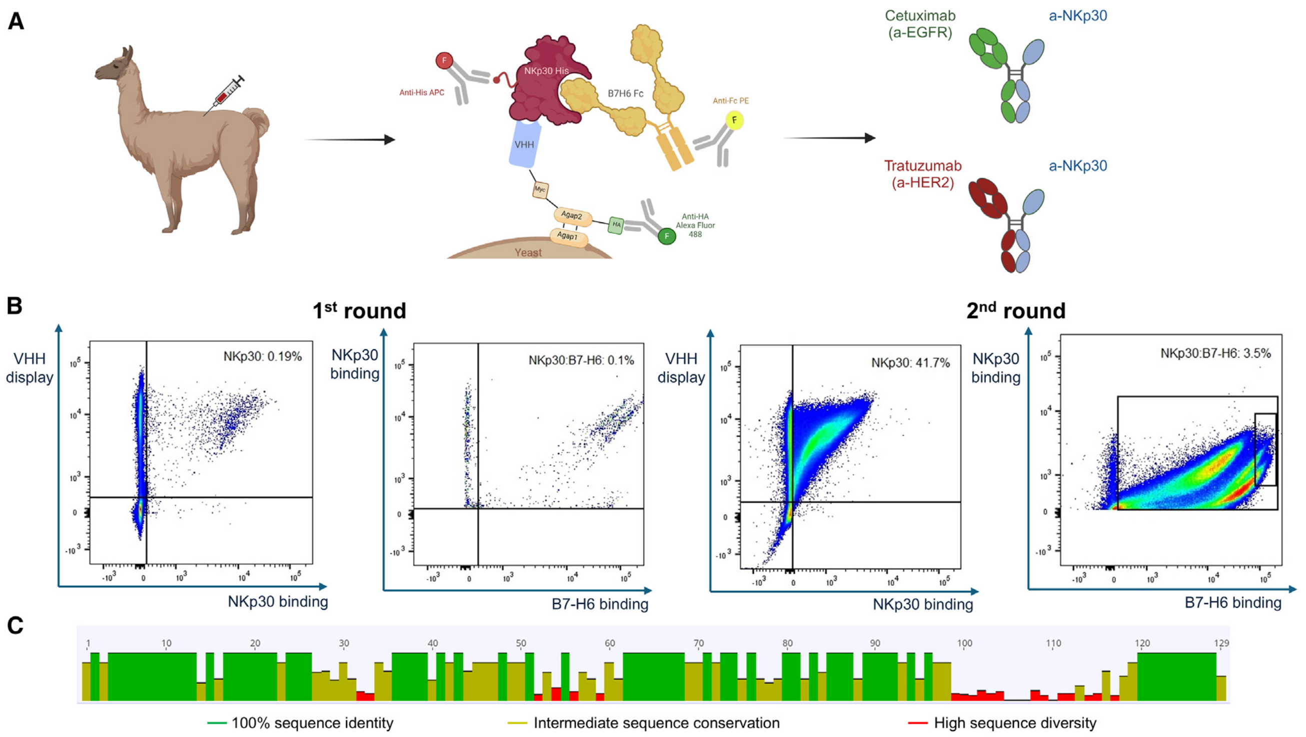

Figure 1. Discovery of non-B7-H6 competitive anti-NKp30 VHHs

(A) Overview of the workflow. Camelids were immunized with NKp30, and a yeast surface display library was constructed. The library was subsequently incubated with NKp30 and B7-H6, as well as their detection antibodies. Selected hits were reformatted as DuoBody with cetuximab arm (anti-EGFR) or trastuzumab arm (anti-HER2). (Created with BioRender.com.) (B) FACS-based discovery of non-B7-H6 competitive VHHs. A three-color-based sorting approach was applied: a two-dimensional gate to identify functional display in combination to NKp30 binding, and then, a second two-dimensional gate was applied to select NKp30 binders that were not competing with B7-H6. From the first round of sorting, 0.19% of the library was binding NKp30, while 0.1% was binding NKp30 in the presence of B7-H6. The 0.1% was selected for sorting. For the second and final round, 41.7% of the library was NKp30 binders. We applied a more stringent sorting gate to select VHHs that were better positively modulating NKp30 affinity for B7-H6 and selected 3.5% of the library for sorting. Applied sorting gates and corresponding cell populations (as percentage of total cells) are shown. Plots were generated using FlowJo. (C) Sequence homology of NKp30 binders. Sequence alignment of NKp30 binders showed a heterogeneous diversity. The alignment was generated with Geneious Prime.

additional positive input for NK cell activation. This natural recognition will complement the induced close proximity facilitated by the NKCE.

In this work, we describe the specific discovery of allosteric anti-NKp30 variable domains of the heavy chain (VHHs) derived from camelid immunization by fluorescence-activated cell sorting (FACS). Based on the DuoBody technology, anti-NKp30 VHH moieties were paired with cetuximab (anti-EGFR) or trastuzumab (anti-human epidermal growth factor receptor 2 [HER2]) to generate bispecific NKCEs. We tested NKCE killing specificity on HeLa cells, a breast cancer cell line expressing both EGFR and B7-H6, or SK-BR-3 cells, a breast cancer cell line expressing both HER2 and B7-H6.

RESULTS

Discovery of non-B7-H6 competitive binders of NKp30

Recently, bispecific antibodies (bsAbs) have been engineered to redirect NK cells closer to tumor cells and engage them via the NKp30 receptor.[17–20] We hypothesized that bsAbs that engage NK cells via NKp30 but do not compete with its ligand B7-H6 will enable better recognition of B7-H6$^+$ tumor cells. This would be due to the natural NKp30-B7-H6 interaction, which triggers an additional activating signal for NK cells. Therefore, we aimed at discovering antibodies specifically targeting allosteric sites of NKp30. To this end, a yeast surface display library was generated from camelid immunization. The diversity of the library was estimated to be 1.5×10^9.

To isolate VHHs that do not compete with B7-H6, we incubated this library with rhNKp30 His-tagged protein and rhB7-H6 biotinylated Fc fusion protein simultaneously to select non-B7-H6 competitive NKp30 binders (Figures 1A and S1).[21] In addition, for the second round, we aimed to refine the selection to VHHs that would positively modulate the affinity of NKp30 for B7-H6 by narrowing the gate to the highest B7-H6 binding fluorescence values, which besides reflecting high affinity for NKp30 would also reflect an increase in B7-H6 binding (Figure 1B). We were able to enrich for non-B7-H6 competitive NKp30 binders within two rounds. Sequencing alignment highlighted nine unique clones from six different clusters based on CDR3

Table 1. Biophysical data of anti-NKp30 antibodies

Antibody	K_D,nM	Bin	Binding to rcNKp30	Binding to rrNKp30
B4	0.667	1	yes	no
B6	35.6	2	yes	yes
B10	4.66	1	yes	no
D10	6.78	1	yes	no
E4	4.32	1	yes	no
E11	6.83	1	yes	yes
G5	7.01	1	yes	yes
G10	2.63	1	yes	yes

Affinities and binding assays were performed by BLI. Recombinant cynomolgus (rc) and recombinant rat (rr).

alignment (Figure 1C). All the sequences were reformatted as DuoBody (F405L) VHH-Fc fusion proteins with Fc effector attenuated mutations (L234A/L235A, LALA)[22,23] and expressed in Expi293F cells. With the exception of one clone that we have not been able to produce, the other variants showed yields in the double to triple milligram per liter scale (Table S1). In addition, aggregation propensities as analyzed by size-exclusion chromatography (SEC) were favorable, with target monomer peaks of more than 90% (Table S1).

Antibodies are binding to two different allosteric epitopes of NKp30

Qualitative binding to rhNKp30 by biolayer interferometry (BLI) revealed that all the expressed antibodies were binding to NKp30. No binding to or competition with B7-H6 was shown. We subsequently determined the binding kinetics to NKp30. In accordance with typical characteristics for antibodies, affinity varied between sub-nanomolar to double-digit nanomolar (Table 1; Figure S2). Then, to scrutinize epitope coverage, we performed an epitope binning assay by pairwise comparison of each variant in every possible orientation, which revealed two epitope bins, with one being dominant (Table 1). This finding is not surprising as the extracellular domain (ECD) of NKp30 is small and may only have a few immunogenic epitopes. Interestingly, antibodies binding to the first bin exhibit higher affinity, from 0.667 nM for B4 to 7.01 nM for G5, while the antibody targeting the second bin, B6, has a 35.6-nM affinity. In addition, we performed a cross-specificity binding assay to related proteins using recombinant human programmed cell death protein-1 (rhPD-1), rhPD-L (ligand) 1, recombinant human cytotoxic T lymphocyte-associated protein 4 (rhCTLA-4), and rhCD28, all of them showing to some extent a structure similar to that of NKp30.[24,25] As expected, no off-target binding was shown (Table 1). We then wanted to confirm the binding to cynomolgus and rat orthologs as NKp30 is a pseudogene in the mouse.[26] While all the antibodies were binding to the cynomolgus protein, only four showed binding to the rat protein (Table 1).

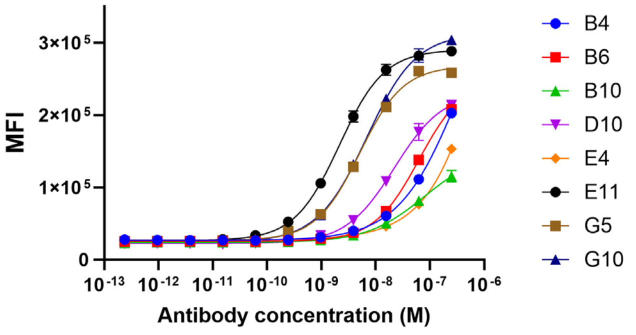

Figure 2. Functional binding assay to NK-92 cells evaluated by FACS
NKp30-expressing NK-92 cells were incubated with decreasing concentrations of anti-NKp30 antibodies. Antibody binding was evaluated via a secondary detection antibody (anti-Fc Alexa Fluor 488 conjugate) on an iQue3.

As a functional binding test, we incubated an immortalized NK cell line (NK-92) expressing NKp30[27] with eight antibodies. Binding to NK-92 cells was confirmed for all antibodies, but a titration assay highlighted different binding profiles (Figure 2). Indeed, antibodies that were not cross-reactive with the rat NKp30 protein showed poor affinity to the endogenous NKp30. The four other antibodies showed comparable binding properties to NKp30 expressing cells regarding BLI values. We hypothesize that antibodies targeting bin 1 are binding to an overlapping epitope that has low similarities between rhNKp30, rrNKp30, and the endogenous NKp30; the involvement of glycans cannot be excluded to explain these differences. Hence, we discontinued the non-rat cross-reactive antibodies for further assays.

Generation of DuoBody

To generate the bsAbs, we relied on the DuoBody technology developed by Genmab, where controlled Fab arm exchange (cFAE) is used to generate bsAbs called DuoBody.[28,29] Our group previously used this technology to generate a functional multispecific DuoBody that can engage NK cells.[30] Hence, we combined the previously described NKp30 binders with cetuximab (NKp30xEGFR) or trastuzumab (NKp30xHER2) that harbor the K409R and L234A/L235A (LALA) mutations. After reoxidation of the DuoBody, we first validated simultaneous binding of the bispecific NKCEs to NKp30 and EGFR (Figure 3A) or HER2 (Figure 3B) by BLI. In addition, we performed a comparable functional assay on HeLa ($EGFR^+$/$B7\text{-}H6^+$) (Figure 3C) or SK-BR-3 ($HER2^+$/$B7\text{-}H6^+$) (Figure 3D) tumor cells.[26,31] Differences in the cellular binding assay can be explained by the fact that HeLa cells express a medium level of EGFR, while SK-BR-3 cells express a high level of HER2.[32,33]

Enhancement of the killing of $B7\text{-}H6^+$/$EGFR^+$ tumor cells via cytokine release

Afterward, the NKp30xEGFR DuoBody constructs were assessed more meticulously for their killing capacities toward HeLa cells using peripheral blood mononuclear cell (PBMC)-isolated NK cells in a dose-response curve (Figure 4A). The killing capacities of NKCEs were compared to the antibody-dependent cell cytotoxicity (ADCC) induced by the commercially approved antibody anti-EGFR antibody cetuximab (Erbitux). All four DuoBody variants triggered robust lysis of HeLa cells in a dose-dependent manner, with potencies (EC_{50} killing) in the two- to three-digit picomolar range (Figure 4B). The

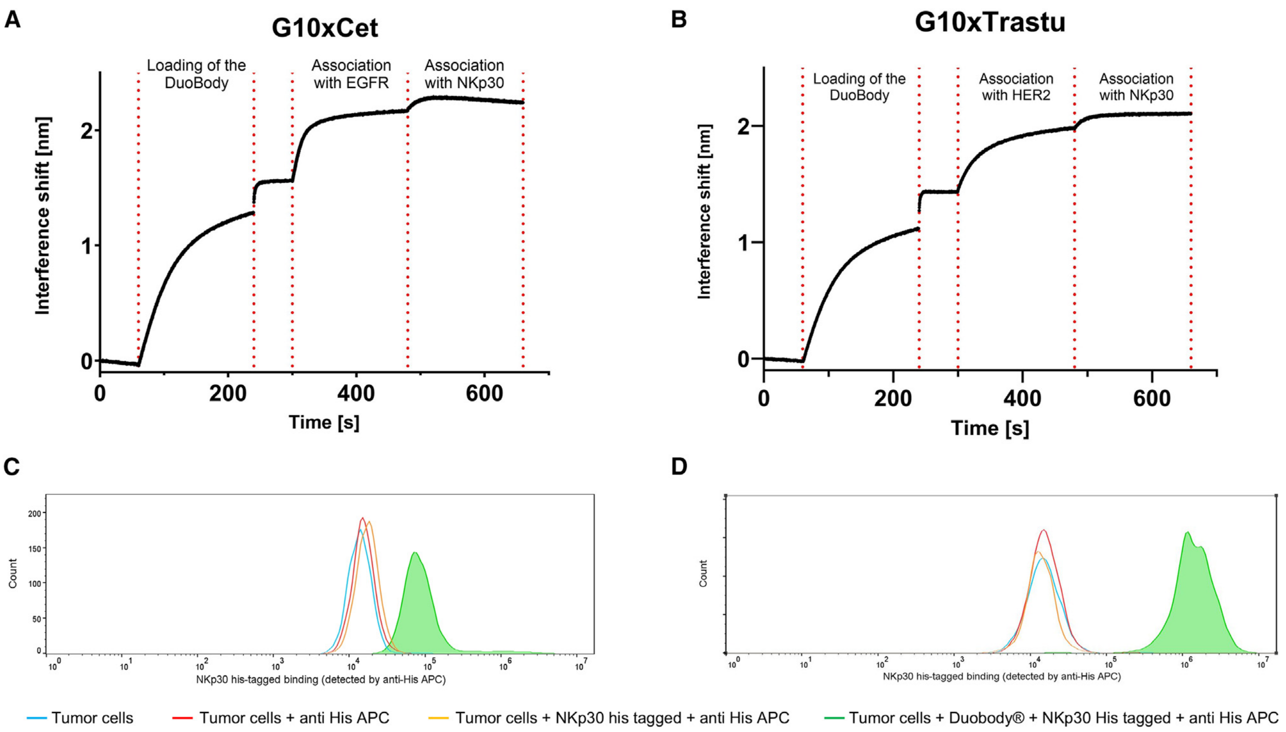

Figure 3. Dual binding of DuoBody determined by BLI and by FACS

G10xcetuximab (A and C) or G10xtrastuzumab (B and D) are shown as examples. (A and B) Dual binding determined by BLI. DuoBody molecules were loaded on AHC2 biosensors. After a baseline step in kinetic buffer, binding to EGFR (A) or HER2 (B) was tested and simultaneous binding capacity to NKp30 was subsequently highlighted. (C and D) Dual binding determined by FACS on tumor cells. HeLa (C) or SK-BR-3 (D) cells were incubated with the corresponding DuoBody and subsequently incubated with NKp30 His-tagged protein. NKp30 His was detected for both constructs, showing simultaneous binding.

most potent construct, B6xcetuximab, has an EC_{50} killing of 47.4 pM, while the least potent was E11xcetuximab, with an EC_{50} killing of 107.8 pM. Potencies were not improved in regard to the control antibody cetuximab (EC_{50} killing: 2.3 pM), but they were slightly improved compared to the B7-H6 competitive cattle-derived NKCE (EC_{50} killing: 150.1 pM). Interestingly, the four DuoBody variants showed significantly improved killing capacity efficacies in comparison with the ADCC effects of cetuximab. Notably, the construct B6xcetuximab exhibits the highest efficacy, at 279.6% (normalized to cetuximab), representing a 2.8-fold change (ratio between a test sample and the control) in killing capacities. The efficacy of the B7-H6 competitive NKCE was 95.51%, representing a fold change of 0.95, which is similar to the value that was determined in our previous study using B7-H6^{-} tumor cells.[20] This shows that the natural recognition of B7-H6 drives the improvement of the killing capacities of non-B7-H6 competitive NKCEs, while the killing capacities of a B7-H6 competitive NKCE remain independent of B7-H6 recognition. No killing of Chinese hamster ovary (CHO) cells (B7-H6^{-}/EGFR^{-}) was observed, proving that killing capacities are restricted to the binding to EGFR-expressing cells indicating tumor target-specific redirection of NK cells.

In addition to NK cell-mediated tumor cell killing, we analyzed the capacity of these DuoBody variants to trigger immunomodulatory interferon-γ (IFN-γ) and tumor necrosis factor α (TNF-α) cytokine release (Figures 4C and 4D). The four NKCEs elicit robust proinflammatory cytokine release for both IFN-γ and TNF-α. As observed in the killing assay, cytokine releases are significantly higher than those induced by cetuximab.

Killing enhancement of NKCE can be translated to B7-H6^{+}/HER2^{+} tumor cells

Subsequently, we wanted to test whether these effects can be translated to another type of NKCE: a bispecific molecule engaging NKp30 and HER2. Thus, we performed the same killing assay with SK-BR-3 cells (B7-H6^{+}/HER2^{+}) and trastuzumab (Herceptin) as a positive control antibody (Figure 5A). Trastuzumab elicits potent ADCC effects in this assay (EC_{50} killing: 7.6 pM), while the control antibody trastuzumab_LALA exhibits a low response as LALA mutations attenuate but do not abolish ADCC, as previously shown.[30] Similarly, but to a lesser extent than the previous observations, the four NKCEs improved the maximal killing capacities of NK cells with limited improvement of their potencies while still being in the picomolar range (Figure 5B). Again, the highest killing efficacy was achieved for the construct harboring the B6 NKp30 targeting moiety at 196% killing (normalized to trastuzumab), representing an

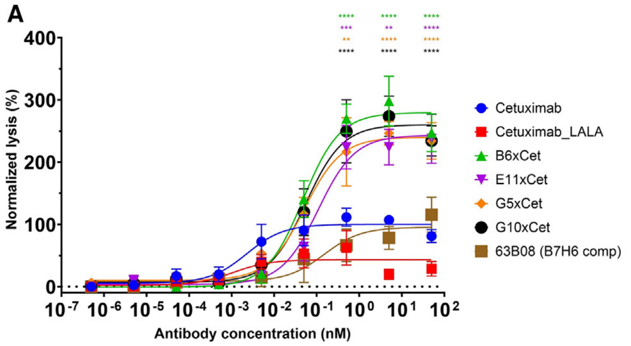

B

Antibody	EC50 killing (pM)	Maximum killing (%)	Fold change
Cetuximab	2.3	100	-
B6xCet	47.4	279.6	2.8
E11xCet	107.8	243.1	2.4
G5xCet	51.5	239.9	2.4
G10xCet	55.4	260.3	2.6
63B08 (B7H6 competitor)	150.1	95.5	0.95
Cetuximab_LALA	1.1	43.3	0.43

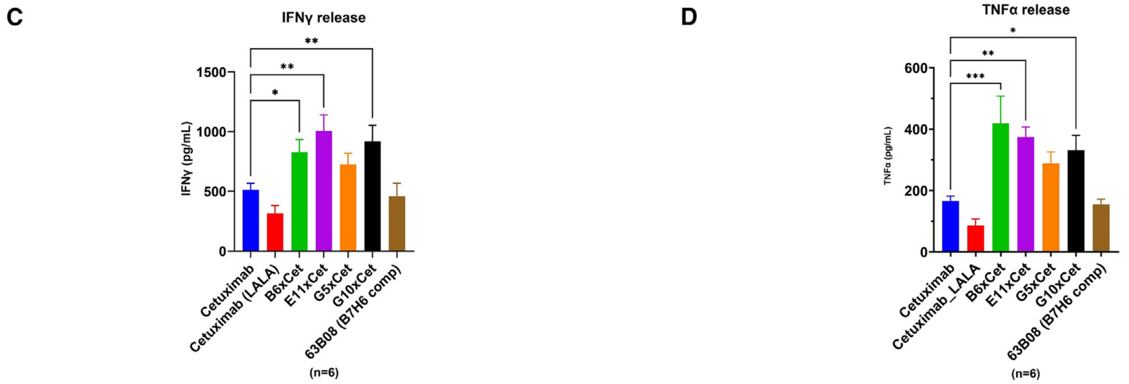

Figure 4. Characterization of NKCE targeting NKp30 and EGFR in terms of killing capacity and cytokine releases on HeLa cells

(A) Fluorescence-based killing assay. HeLa cells were incubated with NK cells at a 1:5 ratio in the presence of the respective NKp30xEGFR targeting NKCE. Cetuximab with or without (LALA) effector-attenuated mutations were used as controls. All data have been normalized to the maximum killing of cetuximab. The graph shows normalized means ± SEMs of $n = 6$ healthy donors. Two-way ANOVA was performed; $^{**}p \leq 0.01$; $^{***}p \leq 0.001$; $^{****}p \leq 0.0001$. (B) NK cell-mediated target cell-dependent cytotoxic properties determined after data fitting. (C) IFN-γ release. NK cell-mediated IFN-γ release was determined by HRTF analysis of the supernatant of the killing assay. The graph shows normalized means ± SEMs of $n = 6$ healthy donors. One-way ANOVA was performed; $^{*}p \leq 0.05$; $^{**}p \leq 0.01$; $^{***}p \leq 0.001$. (D) TNF-α release. NK-cell mediated TNF-α release was determined by HRTF analysis of the supernatant of the killing assay. The graph shows normalized means ± SEMs of $n = 6$ healthy donors. One-way ANOVA was performed; $^{*}p \leq 0.05$; $^{**}p \leq 0.01$; $^{***}p \leq 0.001$.

approximately 2-fold change. No killing of CHO cells (B7-H6^{-}/HER2^{-}) was observed.

DISCUSSION

The focus of this study was to generate potent NKCEs that bridge NKp30 on NK cells with EGFR or HER2 on tumor cells. Our previous studies[18–20] have confirmed that NKp30, an activating receptor expressed on NK cells,[6] can be effectively targeted for the generation of NKCEs in various formats. In these studies, we focused on tumor cells that do not express B7-H6, the natural ligand of NKp30.[9,18–20] Thus, we hypothesized that engineering NKp30-based NKCEs that do not compete with B7-H6 for binding to NKp30 would enable natural tumor recognition via B7-H6, thereby maximizing the effects of the NKCEs.

To achieve this, we applied a discovery strategy based on yeast surface display to obtain VHHs targeting NKp30 that would not compete with the B7-H6 binding site and be suitable for the generation of NKp30-based NKCEs. We employed the DuoBody technology that has been shown to successfully generate bispecific or multispecific antibodies for immune cell redirection.[30]

We engineered DuoBody targeting NKp30xEGFR, which demonstrated a significant enhancement in NK-mediated killing efficacy on HeLa cells compared to the control antibody cetuximab. Additionally, we examined whether these observations could be extended to NKCEs targeting NKp30xHER2, which revealed a slight increase in the killing efficacy of SK-BR-3 cells without increasing the potency.

The architecture of NKCEs is crucial, and we postulate that the different orientation of the tumor-associated targeted antigen moiety may impact the binding to NKp30, allowing for B7-H6 recognition. For instance, trastuzumab targets domain IV of HER2,[34] while cetuximab binds to domain III of EGFR,[35] a more membrane-distal epitope. For T cell engagers, it has been shown that membrane-proximal epitopes are more suitable for immune engagement.[36,37] A systematic approach similar to that made by Chen and colleagues for T cell engagers is needed to fully understand the role of epitope location, target expression, and antibody format.[38] Indeed, HeLa cells

A

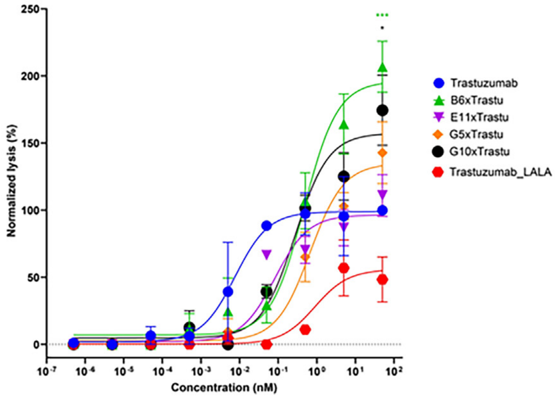

B

Antibody	EC50 killing (pM)	Maximum killing (%)	Fold change
Trastuzumab	7.6	100	-
B6xTrastu	466.6	196	1.9
E11xTrastu	81.5	96.5	0.96
G5xTrastu	671.9	134.8	1.3
G10xTrastu	271.5	157.3	1.6
Trastuzumab_LALA	892.4	55.9	0.56

Figure 5. Killing assay of NKCE targeting NKp30 and HER2 on SK-BR-3 cells

(A) SK-BR-3 cells were incubated with NK cells at a 1:5 ratio in the presence of the respective NKp30xHER2 targeting NKCE. Trastuzumab with or without (LALA) effector-attenuated mutations were used as controls. All data have been normalized to the maximum killing of trastuzumab. The graph shows normalized means ± SEMs of $n = 6$ healthy donors. two-way ANOVA was performed; $^{*}p \leq 0.05$; $^{***}p \leq 0.001$. (B) NK cell-mediated target cell-dependent cytotoxic properties determined after data fitting.

express a higher level of B7-H6 compared to SK-BR-3 cells, which might also account for the enhanced killing capacities observed for EGFR-targeted constructs compared to the HER2-targeted ones.

Here, we propose an enhanced targeting approach for NKCEs, favoring natural B7-H6 recognition. Bivalent targeting has been reported to be advantageous for triggering NK-mediated killing by cross-linking NK surface receptors.[19] Additionally, it would be interesting to evaluate the engineering of antibodies engaging other surface receptors, such as FcγRIIIa.[15,16,18,19,39]

Shedding of B7-H6 by metalloproteases has been reported as an escape mechanism[9,10]; thus, it would be crucial to evaluate these NKCEs *in vivo*. Moreover, it has been shown that NK-mediated killing of CHO cells transfected with B7-H6 was not impacted by non-B7-H6 competitive NKCEs in comparison to their competitive counterparts.[18] This might constitute an advantage for non-B7-H6 competitive NKp30-based NKCEs as they do not interfere with the physiological recognition of B7-H6^{+} cells by NK cells. Nevertheless, for tumor cells that do not express B7-H6, we previously showed that the killing properties are in the range of classical ADCC induction.[18–20] B7-H6 thus represents a favorable biomarker for these non-competitive NKCEs.

In conclusion, the NKCEs generated in this study may represent an interesting NK cell modality for tumor cells harboring B7-H6 and may represent the premise of an enhanced-targeting approach.

MATERIALS AND METHODS

Camelid immunization

One alpaca and one huarizo were immunized using recombinant cynomolgus soluble NKp30 His-tagged protein at Preclinics (Potsdam, Germany). Afterward, total RNA was extracted from PBMCs, and cDNA was synthetized as described in detail elsewhere.[18]

All experimental procedures and animal care were in accordance with European Union animal welfare protection laws and regulations. All animals remained alive after treatment.

Library construction

The library was generated via homologous recombination, as previously described in detail elsewhere.[40,41] Briefly, *Saccharomyces cerevisiae* EBY100 cells were used to generate the yeast surface display library. The camelid VHH repertoire was amplified by PCR introducing homologous regions to the destination vector. Lithium acetate-conditioned cells were resuspended in the electroporation buffer and mixed with the linearized destination vector (pYD), which contains a tryptophan auxotrophic marker, and a pool of the homologous DNA insert. Twelve electroporation reactions were performed in parallel. The library titer was calculated by plating serial dilutions of transformed cells.

Library sorting

Recombinant human His-tagged ECD of NKp30 was purchased from ACROBiosystems and B7-H6 biotinylated Fc fusion protein was purchased from R&D Systems.

For library screening and sorting, yeast cells were grown overnight in SD medium (without tryptophan) for 24 h at 30°C. Afterward, cells were seeded in SG medium (without tryptophan) for 48 h at 20°C.

All labeling steps were performed on ice and in the dark for 30 min with 1×10^7 cells in 20 µL for the controls and 5×10^8 cells in 1 mL for the first sorting round or 1×10^8 cells in 200 µL for the second sorting round. Briefly, cells were first washed with PBS followed by 30 min of incubation simultaneously with NKp30 and B7-H6. Cells were washed twice with PBS and then incubated for 30 min with secondary detection antibodies. VHH surface display was monitored using goat-HA Tag AlexaFluor 488-conjugated antibody (R&D

Systems, 1:20). Detection of antigen binding was performed using SureLight APC Anti-6X His Tag antibody (Abcam, 1:20) for NKp30 His-tagged protein and R-Phycoerythrin AffiniPure Goat Anti-Human IgG, Fc fragment specific (Jackson Immunoresearch, 1:20) for B7-H6 Fc fusion protein. After washing twice with PBS, cells were resuspended in an appropriate volume for FACS sorting on a BD FACSAria Fusion cell sorter (BD Biosciences). After enrichment, yeast plasmids were isolated using the MasterPure Yeast DNA purification kit (Biosearch Technologies). *Escherichia coli* cells were transformed with the isolated yeast plasmids by electroporation. After overnight growth on agar plates with ampicillin as a selection marker, 96 clones were randomly selected for Sanger sequencing at Microsynth.

Expression and purification

After sequencing, selected hits were reformatted into a pTT5 plasmid as DuoBody (F405L) VHH-Fc fusion protein, with effector attenuated mutations (L234A/L235A, LALA) by Geneart (Thermo Fisher Scientific). Cetuximab and trastuzumab were reformatted into a PTT5 plasmid as DuoBody (K409R) and effector-attenuated mutations (LALA) also by Geneart (Thermo Fisher Scientific). For expression, Expi293F cells were transiently transfected with expression vectors according to the manufacturer's protocol (Thermo Fisher Scientific). Harvest of the cells was done 7 days post-transfection and antibodies were purified using MabSelect antibody purification chromatography resin (GE Healthcare). Samples were formulated in PBS and concentrations were determined using the QIA expert system (Qiagen) after sterile filtration with Ultrafree-CL centrifugal devices (Merck Millipore). Purity and aggregate formation were evaluated by SEC analysis.

cFAE

cFAE was performed according to an optimized protocol that our group recently described elsewhere[42] by manual pipetting in a 24-well deep-well plates at room temperature with a 1-mL final volume of DuoBody products. Parental antibodies were normalized to 14 μM and combined to a 1:1 M ratio. Subsequently, a 20-fold molar excess of Tris(2-carboxyethyl)phosphine (Sigma-Aldrich) was added for an incubation time of 6 h. Afterward, a 40-M excess of PEG-azide (Sigma-Aldrich) was added for quenching, followed by 6 days of incubation at room temperature to allow reoxidation of the samples.

BLI

All the experiments have been performed using the Octet RED BLI system (Sartorius) according to the manufacturer's guidelines at 25°C with 1,000 rpm agitation. Relevant controls were included for each experiment (e.g., unloaded biosensor, unrelated antigen).

For the initial qualitative binding assay, antibodies were loaded onto anti-human IgG Fc capture (AHC2) biosensors for 3 min (5 μg/mL), after a 1-min baseline step in kinetic buffer (KB; PBS +0.1% Tween 20 + 1% BSA) association to rhNKp30 was tested for 3 min followed by a dissociation step in KB for 3 min.

For kinetic analysis, NKp30 binders were loaded on AHC2 biosensors. Association with decreasing concentrations of the NKp30 was measured for 5 min, followed by a dissociation step for 10 min in KB. Data were analyzed using Sartorius analysis software and dissociation constant (K_D) values were determined by fitting to a 1:1 model.

Competition/epitope binning assays were performed by coupling the antibodies (5 μg/mL, in acetate buffer, pH 6.0) on activated ARG2G biosensors, after quenching (1 M ethanolamine, pH 8.5); a rinsing step in KB lasted for 1 min. The first association with NKp30 lasted for 3 min, followed by another association for 3 min with B7-H6 or the aforementioned NKp30 binders. For each molecule, both orientations were tested to highlight overlapping epitopes.

For the cross-reactivity binding assay, we used rhNKp46 and rhCTLA-4 (ACROBiosystems), rhPD-1 (Sino Biological), rhPD-L1, recombinant cynomolgus monkey NKp30, and recombinant rat NKp30 (R&D Systems). Antibodies were loaded on AHC biosensors (5 μg/mL) for 3 min, and binding was tested by association/dissociation of the cross-reactive protein (100 nM) for 3 min.

For analyzing simultaneous binding, the DuoBody antibodies were loaded onto AHC2 sensors (5 μg/mL) for 3 min. Afterward, a first association step was performed using 100 nM rhEGFR (produced by Merck Healthcare KGaA), or alternatively, 100 nM rhHER2 (Sino Biological) for 3 min, followed by a second association with 100 nM rhNKp30 for 3 min.

Functional binding assays

NKp30-expressing NK-92 cells were cultured in MEM alpha medium supplemented with 12.5% heat-inactivated fetal bovine serum (FBS), 2.5% horse serum, and 2 mM glutamine. HeLa cells were cultured in DMEM, and SK-BR-3 cells were cultured in McCoy's 5A medium; both were supplemented with 10% FBS and 2 mM glutamine. Cells were cultivated in a CO_2-humidified incubator at 37°C. Functional binding of the expressed antibodies on NKp30-expressing cells was evaluated using an iQue3 (Sartorius).

As an initial binding test, 10^5 NK-92 cells/well were seeded, and after two washing steps in PBS + 1% BSA, they were incubated on ice for 1 h with 100 nM of antibodies. After two washing steps, 250 nM of Alexa Fluor 488 anti-human Fc (Jackson Immunoresearch) was used for staining for another 30 min on ice and in the dark. After two washing steps, 20 μg/mL propidium iodide (Invitrogen) was used to label dead cells in a total volume of 100 μL. Relevant controls were included and gates adjusted according to these controls (e.g., no antibody or detection antibody only). A titration of anti-NKp30 binders on NK-92 cells has been done following the aforementioned protocol, except for the antibody concentration (serial dilution).

Simultaneous binding of DuoBody was also tested by flow cytometry, as we described previously.[20] Briefly, HeLa cells (NKp30xEGFR constructs) or SK-BR-3 cells (NKp30xHER2 constructs) were seeded and

incubated with 100 nM DuoBody for 1 h at 4°C. After two washing steps, they were incubated with 200 nM NKp30 His-tagged protein for 30 min at 4°C, subsequently washed twice and incubated with anti-His Alexa Fluor 488 for 30 min at 4°C. After two final washing steps cells were resuspended with PBS + 1% BSA, 20 μg/mL propidium iodide.

Killing assay

All the experiments have been performed in accordance with the Declaration of Helsinki. PBMCs were isolated from the blood of healthy donors by density gradient centrifugation prior to NK cell isolation, after receiving written informed consent. NK cells were isolated from PBMCs using the EasySep Human NK Cell Isolation Kit (STEMCELL Technologies) on the RoboSep-S device and were subsequently cultivated overnight in complete medium (AIM V) supplemented with interleukin-2 (100 U/mL, ACROBiosystems). The next day, the concentration of NK cells was adjusted at 0.625 × 10^6 cells/mL. HeLa, SK-BR-3, and ExpiCHO cells as negative control were stained with CellTracker Deep Red Dye (Thermo Fisher Scientific). A total of 2,500 cells/well were seeded into a 384-well clear-bottom culture plate and subsequently incubated at 37°C. After adherence, NK cells were dispensed in the plate along with the indicated concentration of DuoBody (the ratio of effector cells to target cells was 5:1). In addition, 0.03 μM SYTOX Green Dead Cell Stain (Invitrogen) was added to each well to detect dead cells. Cetuximab (Merck Healthcare KGaA) and trastuzumab (Roche) with effector-competent functions were used as positive controls. Fluorescence (red and green) was monitored over 24 h by incubation in the Incucyte system (Sartorius). After subtraction of basal killing (control with no antibody treatment), the percentage of lysis was calculated as follows: (overlay fluorescence of green + red area)/(fluorescence of red area) × 100.

Cytokine release assays

Supernatants from the aforementioned assay were collected and analyzed utilizing the human IFN-γ and TNF-α Homogeneous Time-Resolved Fluorescence (HTRF) kits from Revvity following the manufacturer's instructions. Plates were measured with PHERAstar FSX (BMG Labtech), and data were analyzed using MARS software enabling a four-parameter model fitting of the standard curve.

Data analysis

FlowJo software was used for plotting FACS data. Other graphical and statistical analysis were performed with GraphPad Prism 8 software. When ANOVA was performed, $p \leq 0.05$ was regarded as significant.

DATA AND CODE AVAILABILITY

The data presented in this study are available upon reasonable request to the corresponding author.

ACKNOWLEDGMENTS

The authors thank Sigrid Auth and Nils Bahl for experimental support. This project received funding from the European Union's Horizon 2020 research and innovation programme under the Marie Skłodowska-Curie grant agreement no. 956314 (ALLODD).

AUTHOR CONTRIBUTIONS

Conceptualization, L.F., L.P., and S.Z. Methodology, L.F. Investigation, L.F. and P.A. Data curation, L.F. Writing – original draft, L.F. Writing – review & editing, P.A., L.P., H.K., S.Z., L.T., and S.B. Resources, P.A., L.P., and S.Z. Supervision, H.K. and S.B. Project administration, L.T. and S.B. Funding acquisition, L.T. and S.B.

DECLARATION OF INTERESTS

L.F., P.A., L.P., S.Z., L.T., and S.B. are employees of Merck Healthcare KGaA.

SUPPLEMENTAL INFORMATION

Supplemental information can be found online at https://doi.org/10.1016/j.omton.2024.200917.

REFERENCES

1. Rebuffet, L., Melsen, J.E., Escalière, B., Basurto-Lozada, D., Bhandoola, A., Björkström, N.K., Bryceson, Y.T., Castriconi, R., Cichocki, F., Colonna, M., et al. (2024). High-dimensional single-cell analysis of human natural killer cell heterogeneity. Nat. Immunol. *25*, 1474–1488. https://doi.org/10.1038/s41590-024-01883-0.

2. Vivier, E., Tomasello, E., Baratin, M., Walzer, T., and Ugolini, S. (2008). Functions of natural killer cells. Nat. Immunol. *9*, 503–510. https://doi.org/10.1038/ni1582.

3. Quatrini, L., Della Chiesa, M., Sivori, S., Mingari, M.C., Pende, D., and Moretta, L. (2021). Human NK cells, their receptors and function. Eur. J. Immunol. *51*, 1566–1579. https://doi.org/10.1002/eji.202049028.

4. Barrow, A.D., Martin, C.J., and Colonna, M. (2019). The Natural Cytotoxicity Receptors in Health and Disease. Front. Immunol. *10*, 909. https://doi.org/10.3389/fimmu.2019.00909.

5. Koch, J., Steinle, A., Watzl, C., and Mandelboim, O. (2013). Activating natural cytotoxicity receptors of natural killer cells in cancer and infection. Trends. Immunol. *34*, 182–191. https://doi.org/10.1016/j.it.2013.01.003.

6. Pende, D., Parolini, S., Pessino, A., Sivori, S., Augugliaro, R., Morelli, L., Marcenaro, E., Accame, L., Malaspina, A., Biassoni, R., et al. (1999). Identification and Molecular Characterization of Nkp30, a Novel Triggering Receptor Involved in Natural Cytotoxicity Mediated by Human Natural Killer Cells. J. Exp. Med. *190*, 1505–1516. https://doi.org/10.1084/jem.190.10.1505.

7. Brandt, C.S., Baratin, M., Yi, E.C., Kennedy, J., Gao, Z., Fox, B., Haldeman, B., Ostrander, C.D., Kaifu, T., Chabannon, C., et al. (2009). The B7 family member B7-H6 is a tumor cell ligand for the activating natural killer cell receptor NKp30 in humans. J. Exp. Med. *206*, 1495–1503. https://doi.org/10.1084/jem.20090681.

8. Pazina, T., Shemesh, A., Brusilovsky, M., Porgador, A., and Campbell, K.S. (2017). Regulation of the Functions of Natural Cytotoxicity Receptors by Interactions with Diverse Ligands and Alterations in Splice Variant Expression. Front. Immunol. *8*, 369. https://doi.org/10.3389/fimmu.2017.00369.

9. Schlecker, E., Fiegler, N., Arnold, A., Altevogt, P., Rose-John, S., Moldenhauer, G., Sucker, A., Paschen, A., von Strandmann, E.P., Textor, S., and Cerwenka, A. (2014). Metalloprotease-Mediated Tumor Cell Shedding of B7-H6, the Ligand of the Natural Killer Cell–Activating Receptor NKp30. Cancer. Res. *74*, 3429–3440. https://doi.org/10.1158/0008-5472.CAN-13-3017.

10. Pesce, S., Tabellini, G., Cantoni, C., Patrizi, O., Coltrini, D., Rampinelli, F., Matta, J., Vivier, E., Moretta, A., Parolini, S., and Marcenaro, E. (2015). B7-H6-mediated downregulation of NKp30 in NK cells contributes to ovarian carcinoma immune escape. OncoImmunology *4*, e1001224. https://doi.org/10.1080/2162402X.2014.1001224.

11. Dianat-Moghadam, H., Rokni, M., Marofi, F., Panahi, Y., and Yousefi, M. (2018). Natural killer cell–based immunotherapy: From transplantation toward targeting cancer stem cells. J. Cell. Physiol. *234*, 259–273. https://doi.org/10.1002/jcp.26878.

12. Vivier, E., Rebuffet, L., Narni-Mancinelli, E., Cornen, S., Igarashi, R.Y., and Fantin, V.R. (2024). Natural killer cell therapies. Nature *626*, 727–736. https://doi.org/10.1038/s41586-023-06945-1.

13. Demaria, O., Gauthier, L., Debroas, G., and Vivier, E. (2021). Natural killer cell engagers in cancer immunotherapy: Next generation of immuno-oncology treatments. Eur. J. Immunol. *51*, 1934–1942. https://doi.org/10.1002/eji.202048953.

14. Demaria, O., Habif, G., Le Floch, F., Chiossone, L., Remark, R., Vetizou, M., Maurel, N., Gauthier, L., Morel, Y., Paturel, C., et al. (2022). IPH6501 Is a Novel NKp46-Targeting Tetraspecific Antibody-Based Natural Killer Cell Engager Therapeutic (ANKET) Armed with a Non-Alpha IL-2 Variant and Developed for the Treatment of CD20-Positive Malignancies. Blood *140*, 11559. https://doi.org/10.1182/blood-2022-163561.

15. Lipinski, B., Arras, P., Pekar, L., Klewinghaus, D., Boje, A.S., Krah, S., Zimmermann, J., Klausz, K., Peipp, M., Siegmund, V., et al. (2023). NKp46-specific single domain antibodies enable facile engineering of various potent NK cell engager formats. Protein. Sci. *32*, e4593. https://doi.org/10.1002/pro.4593.

16. Gauthier, L., Morel, A., Anceriz, N., Rossi, B., Blanchard-Alvarez, A., Grondin, G., Trichard, S., Cesari, C., Sapet, M., Bosco, F., et al. (2019). Multifunctional Natural Killer Cell Engagers Targeting NKp46 Trigger Protective Tumor Immunity. Cellule *177*, 1701–1713.e16. https://doi.org/10.1016/j.cell.2019.04.041.

17. Peipp, M., Derer, S., Lohse, S., Staudinger, M., Klausz, K., Valerius, T., Gramatzki, M., and Kellner, C. (2015). HER2-specific immunoligands engaging NKp30 or NKp80 trigger NK-cell-mediated lysis of tumor cells and enhance antibody-dependent cell-mediated cytotoxicity. Oncotarget *6*, 32075–32088. https://doi.org/10.18632/oncotarget.5135.

18. Klausz, K., Pekar, L., Boje, A.S., Gehlert, C.L., Krohn, S., Gupta, T., Xiao, Y., Krah, S., Zaynagetdinov, R., Lipinski, B., et al. (2022). Multifunctional NK Cell–Engaging Antibodies Targeting EGFR and NKp30 Elicit Efficient Tumor Cell Killing and Proinflammatory Cytokine Release. J. Immunol. *209*, 1724–1735. https://doi.org/10.4049/jimmunol.2100970.

19. Boje, A.S., Pekar, L., Koep, K., Lipinski, B., Rabinovich, B., Evers, A., Gehlert, C.L., Krohn, S., Xiao, Y., Krah, S., et al. (2024). Impact of antibody architecture and paratope valency on effector functions of bispecific NKp30 x EGFR natural killer cell engagers. mAbs *16*, 2315640. https://doi.org/10.1080/19420862.2024.2315640.

20. Klewinghaus, D., Pekar, L., Arras, P., Krah, S., Valldorf, B., Kolmar, H., and Zielonka, S. (2021). Grabbing the Bull by Both Horns: Bovine Ultralong CDR-H3 Paratopes Enable Engineering of "Almost Natural" Common Light Chain Bispecific Antibodies Suitable For Effector Cell Redirection. Front. Immunol. *12*, 801368. https://doi.org/10.3389/fimmu.2021.801368.

21. Fournier, L., Demir, D., Elter, D., Pekar, L., Kolmar, H., Toleikis, L., and Becker, S. (2024). A platform for the early selection of non-competitive antibody-fragments from yeast surface display libraries. Biol. Chem. https://doi.org/10.1515/hsz-2024-0102.

22. Lund, J., Winter, G., Jones, P.T., Pound, J.D., Tanaka, T., Walker, M.R., Artymiuk, P.J., Arata, Y., Burton, D.R., Jefferis, R., et al. (1991). Human Fc gamma RI and Fc gamma RII interact with distinct but overlapping sites on human IgG. J. Immunol. *147*, 2657–2662. https://doi.org/10.4049/jimmunol.147.8.2657.

23. Wines, B.D., Powell, M.S., Parren, P.W., Barnes, N., and Hogarth, P.M. (2000). The IgG Fc Contains Distinct Fc Receptor (FcR) Binding Sites: The Leukocyte Receptors FcγRI and FcγRIIa Bind to a Region in the Fc Distinct from That Recognized by Neonatal FcR and Protein A1. J. Immunol. *164*, 5313–5318. https://doi.org/10.4049/jimmunol.164.10.5313.

24. Li, Y., Wang, Q., and Mariuzza, R.A. (2011). Structure of the human activating natural cytotoxicity receptor NKp30 bound to its tumor cell ligand B7-H6. J. Exp. Med. *208*, 703–714. https://doi.org/10.1084/jem.20102548.

25. Joyce, M.G., Tran, P., Zhuravleva, M.A., Jaw, J., Colonna, M., and Sun, P.D. (2011). Crystal structure of human natural cytotoxicity receptor NKp30 and identification of its ligand binding site. Proc. Natl. Acad. Sci. USA *108*, 6223–6228. https://doi.org/10.1073/pnas.1100622108.

26. Bjørnsen, E.G., Thiruchelvam-Kyle, L., Hoelsbrekken, S.E., Henden, C., Saether, P.C., Boysen, P., Daws, M.R., and Dissen, E. (2019). B7H6 is a functional ligand for NKp30 in rat and cattle and determines NKp30 reactivity toward human cancer cell lines. Eur. J. Immunol. *49*, 54–65. https://doi.org/10.1002/eji.201847746.

27. Kotzur, R., Duev-Cohen, A., Kol, I., Reches, A., Mandelboim, O., and Stein, N. (2022). NK-92 cells retain vitality and functionality when grown in standard cell culture conditions. PLoS One *17*, e0264897. https://doi.org/10.1371/journal.pone.0264897.

28. Labrijn, A.F., Meesters, J.I., De Goeij, B.E.C.G., Van Den Bremer, E.T.J., Neijssen, J., Van Kampen, M.D., Strumane, K., Verploegen, S., Kundu, A., Gramer, M.J., et al. (2013). Efficient generation of stable bispecific IgG1 by controlled Fab-arm exchange. Proc. Natl. Acad. Sci. *110*, 5145–5150. https://doi.org/10.1073/pnas.1220145110.

29. Labrijn, A.F., Meesters, J.I., Priem, P., de Jong, R.N., van den Bremer, E.T.J., van Kampen, M.D., Gerritsen, A.F., Schuurman, J., and Parren, P.W.H.I. (2014). Controlled Fab-arm exchange for the generation of stable bispecific IgG1. Nat. Protoc. *9*, 2450–2463. https://doi.org/10.1038/nprot.2014.169.

30. Yanakieva, D., Pekar, L., Evers, A., Fleischer, M., Keller, S., Mueller-Pompalla, D., Toleikis, L., Kolmar, H., Zielonka, S., and Krah, S. (2022). Beyond bispecificity: Controlled Fab arm exchange for the generation of antibodies with multiple specificities. mAbs *14*, 2018960. https://doi.org/10.1080/19420862.2021.2018960.

31. Banu, N., Riera-Leal, A., Haramati, J., Ortiz-Lazareno, P.C., Panikar, S.S., Bastidas-Ramirez, B.E., Gutierrez-Silerio, G.Y., Solorzano-Ibarra, F., Tellez-Bañuelos, M.C., Gutierrez-Franco, J., et al. (2020). B7-H6, an immunoligand for the natural killer cell activating receptor NKp30, reveals inhibitory effects on cell proliferation and migration, but not apoptosis, in cervical cancer derived-cell lines. BMC. Cancer. *20*, 1083. https://doi.org/10.1186/s12885-020-07608-4.

32. Zhang, F., Wang, S., Yin, L., Yang, Y., Guan, Y., Wang, W., Xu, H., and Tao, N. (2015). Quantification of Epidermal Growth Factor Receptor Expression Level and Binding Kinetics on Cell Surfaces by Surface Plasmon Resonance Imaging. Anal. Chem. *87*, 9960–9965. https://doi.org/10.1021/acs.analchem.5b02572.

33. Chung, I., Reichelt, M., Shao, L., Akita, R.W., Koeppen, H., Rangell, L., Schaefer, G., Mellman, I., and Sliwkowski, M.X. (2016). High cell-surface density of HER2 deforms cell membranes. Nat. Commun. *7*, 12742. https://doi.org/10.1038/ncomms12742.

34. Cho, H.-S., Mason, K., Ramyar, K.X., Stanley, A.M., Gabelli, S.B., Denney, D.W., and Leahy, D.J. (2003). Structure of the extracellular region of HER2 alone and in complex with the Herceptin Fab. Nature *421*, 756–760. https://doi.org/10.1038/nature01392.

35. Li, S., Schmitz, K.R., Jeffrey, P.D., Wiltzius, J.J.W., Kussie, P., and Ferguson, K.M. (2005). Structural basis for inhibition of the epidermal growth factor receptor by cetuximab. Cancer Cell *7*, 301–311. https://doi.org/10.1016/j.ccr.2005.03.003.

36. Bluemel, C., Hausmann, S., Fluhr, P., Sriskandarajah, M., Stallcup, W.B., Baeuerle, P.A., and Kufer, P. (2010). Epitope distance to the target cell membrane and antigen size determine the potency of T cell-mediated lysis by BiTE antibodies specific for a large melanoma surface antigen. Cancer. Immunol. Immunother. *59*, 1197–1209. https://doi.org/10.1007/s00262-010-0844-y.

37. Li, J., Stagg, N.J., Johnston, J., Harris, M.J., Menzies, S.A., DiCara, D., Clark, V., Hristopoulos, M., Cook, R., Slaga, D., et al. (2017). Membrane-Proximal Epitope Facilitates Efficient T Cell Synapse Formation by Anti-FcRH5/CD3 and Is a Requirement for Myeloma Cell Killing. Cancer Cell *31*, 383–395. https://doi.org/10.1016/j.ccell.2017.02.001.

38. Chen, W., Yang, F., Wang, C., Narula, J., Pascua, E., Ni, I., Ding, S., Deng, X., Chu, M.L.-H., Pham, A., et al. (2021). One size does not fit all: navigating the multi-dimensional space to optimize T-cell engaging protein therapeutics. mAbs *13*, 1871171. https://doi.org/10.1080/19420862.2020.1871171.

39. Colomar-Carando, N., Gauthier, L., Merli, P., Loiacono, F., Canevali, P., Falco, M., Galaverna, F., Rossi, B., Bosco, F., Caratini, M., et al. (2022). Exploiting Natural Killer Cell Engagers to Control Pediatric B-cell Precursor Acute Lymphoblastic Leukemia. Cancer Immunol. Res. *10*, 291–302. https://doi.org/10.1158/2326-6066.CIR-21-0843.

40. Benatuil, L., Perez, J.M., Belk, J., and Hsieh, C.-M. (2010). An improved yeast transformation method for the generation of very large human antibody libraries. Protein Eng. Des. Sel. *23*, 155–159. https://doi.org/10.1093/protein/gzq002.

41. Roth, L., Krah, S., Klemm, J., Günther, R., Toleikis, L., Busch, M., Becker, S., and Zielonka, S. (2020). Isolation of Antigen-Specific VHH Single-Domain Antibodies by Combining Animal Immunization with Yeast Surface Display. Methods. Mol. Biol. *2070*, 173–189. https://doi.org/10.1007/978-1-4939-9853-1_10.

42. Barron, N., Dickgiesser, S., Fleischer, M., Bachmann, A.-N., Klewinghaus, D., Hannewald, J., Ciesielski, E., Kusters, I., Hammann, T., Krause, V., et al. (2024). A Generic Approach for Miniaturized Unbiased High-Throughput Screens of Bispecific Antibodies and Biparatopic Antibody–Drug Conjugates. Int. J. Mol. Sci. *25*, 2097. https://doi.org/10.3390/ijms25042097.

OMTON, Volume *33*

Supplemental information

Enhancing NK cell-mediated tumor killing of B7-H6^{+} cells with bispecific antibodies targeting allosteric sites of NKp30

Léxane Fournier, Paul Arras, Lukas Pekar, Harald Kolmar, Stefan Zielonka, Lars Toleikis, and Stefan Becker

Table S1: Analytics and Yields of a-NKp30 VHH-Fc fusion. Expression yields were determined after protein purification. Purity was determined by analytic size-exclusion chromatography (SEC). HEK: Human Embryonic Kidney. CHO: Chinese Hamster Ovaries. ND: non-determined

Antibody	Yield HEK (mg/L)	SEC HEK (%)	Yield CHO (mg/L)	SEC CHO (%)
A10	ND	ND	ND	ND
B4	220.5	100	201.1	95.5
B6	121.4	98.8	39.7	91.1
B10	141.6	77.5	47.5	96.7
D10	211.9	98.9	210.1	90.2
E4	268.5	100	242.3	100
E11	287.6	100	297.5	100
G5	271.9	100	249.8	100
G10	122.1	100	54.8	100

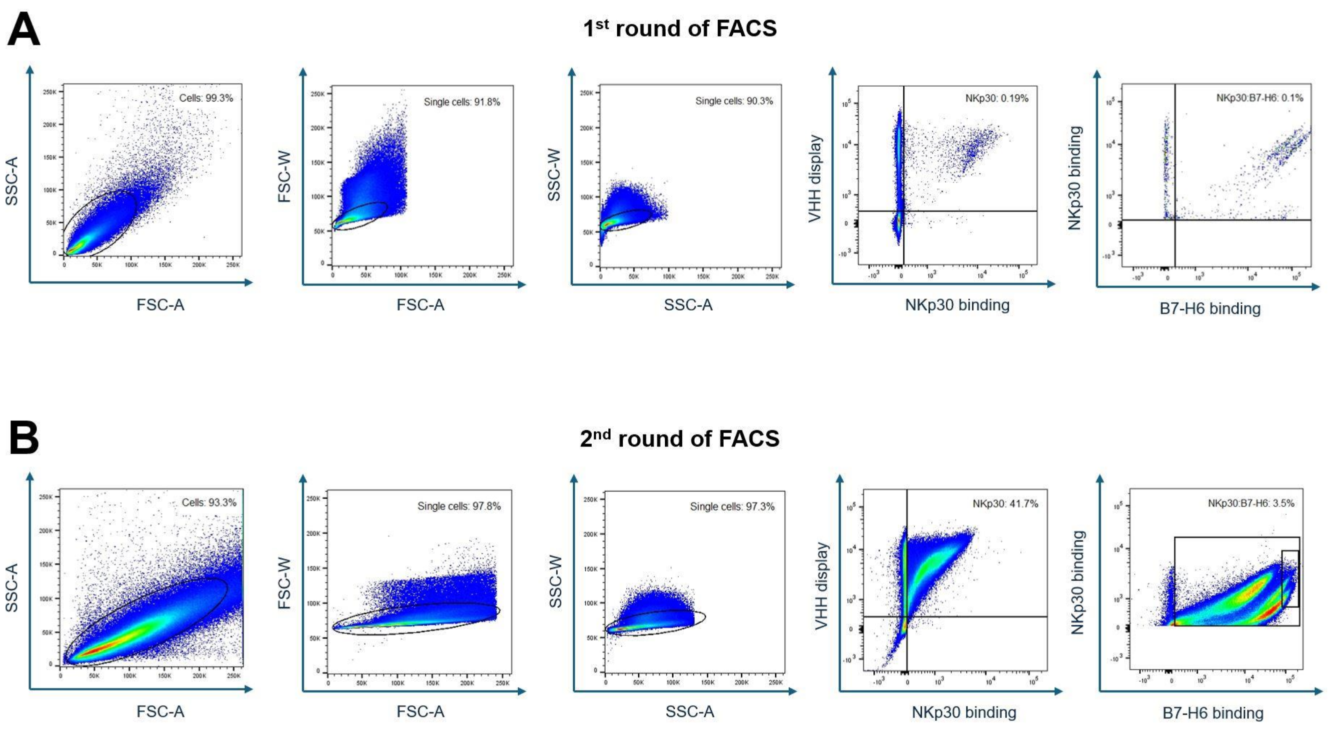

Figure S1: Gating strategy for the sorting of non-B7-H6 competitive NKp30-binders.

The yeast surface display library was screened by FACS. The first gate (FSC-A vs SSC-A) was designed to select yeast cells. From these cells, we selected single cells first based on the plot FSC-A vs FSC-W, and secondly from a gate SSC-A vs SSC-W. From these single cells, a two-dimensional gate to identify functional VHH display in combination to NKp30 binding. From these NKp30 binders, a second two-dimensional gate was applied to select NKp30 binders that were not competing with B7-H6. From the first round of sorting 0.19% of the library were binding NKp30 while 0.1 % were binding NKp30 in the presence of B7-H6. These 0.1% were selected for sorting. For the second and final round, 41.7% of the library were NKp30 binders. We applied a more stringent sorting gate to select VHHs that were better positively modulating NKp30 affinity for B7-H6 and selected 3.5% of the library for sorting.

Applied sorting gates and corresponding cell populations (as % of total cells) are shown. Plots were generated using FlowJo™.

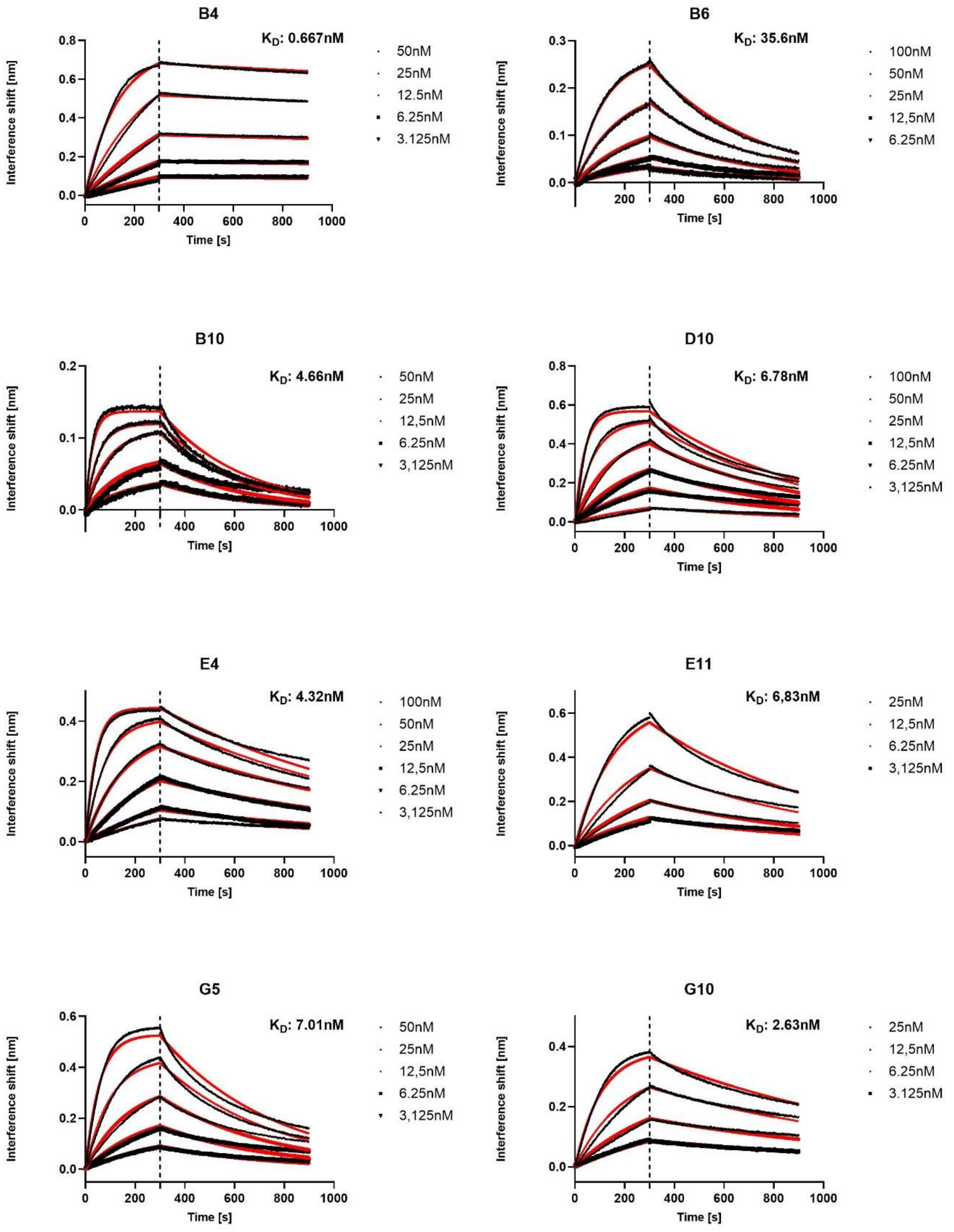

Figure S2: K_D determination of anti-NKp30 for rhNKp30. The experiment was performed by BLI. Data were fitted to a 1:1 model (fitting in red).
